# The dual nature of immunotherapy in female reproductive disorders: immune homeostasis and clinical challenges

**DOI:** 10.3389/fimmu.2025.1658681

**Published:** 2025-10-02

**Authors:** Yiliminuer Abulajiang, Yumei Wu, Yue He

**Affiliations:** ^1^ Beijing Obstetrics and Gynecology Hospital, Capital Medical University, Beijing Maternal and Child Health Care Hospital, Beijing, China; ^2^ Department of Immunology, School of Basic Medical Sciences, Laboratory for Clinical Medicine, Capital Medical University, Beijing, China

**Keywords:** immune checkpoint inhibitors, reproductive toxicity, gynecologic malignancies, immune homeostasis, fertility preservation

## Abstract

The female reproductive system (FRS) exhibits unique immunological characteristics, balancing defense against pathogens with tolerance to sperm and semi-allogeneic embryos. Key players include decidual natural killer (dNK) cells, immune checkpoint molecules (ICMs) and a complex immune microenvironment (IME). Dysregulation of these elements contributes to diseases like recurrent spontaneous abortion (RSA), endometriosis, primary ovarian insufficiency (POI), and infertility. Immunotherapy, particularly immune checkpoint inhibitors (ICIs) and chimeric antigen receptor (CAR) T-cell therapy, shows significant promise in treating gynecological malignancies (e.g., cervical, endometrial, ovarian cancers), especially in advanced/recurrent settings or with specific biomarkers like mismatch repair deficiency. However, challenges persist, including limited efficacy in microsatellite stable tumors, resistance mechanisms and significant immune-related adverse events (irAEs). Critically, emerging evidence indicates potential detrimental effects of immunotherapy (especially ICIs) on female reproductive function, including diminished ovarian reserve, impaired oocyte maturation, hormonal disruption, and possible infertility, mediated by inflammatory responses, gonadotoxicity, and disruption of immune tolerance. Management of female-specific toxicities requires personalized strategies, fertility assessment, and consideration of preservation techniques. Future directions emphasize the development of predictive biomarkers, optimization of combination therapies, and implementation of truly individualized treatment regimens that account for the unique FRS IME, sex hormone influences, and the imperative to preserve fertility. Addressing the reproductive toxicity of novel immunotherapies remains a critical unmet research need.

## 1 Introduction

The FRS maintains a unique immune dichotomy: defending against pathogens while establishing active immune tolerance to allogeneic sperm and semi-allogeneic embryos. This equilibrium relies on dNK cell-mediated fetal-maternal exemption, immunosuppression by regulatory T (Treg) cells, and dynamic expression of ICMs (1, 2). Disruption of this immune homeostasis directly contributes to reproductive pathologies such as RSA and endometriosis (3). ICIs have achieved breakthrough efficacy in gynecologic malignancies, inducing durable remission in mismatch repair-deficient cervical and endometrial cancers (4). However, microsatellite-stable ovarian cancer exhibits limited response to ICIs due to immunosuppressive tumor microenvironment (TME) and intrinsic resistance, highlighting an urgent need for improved therapeutic strategies (5). Notably, ICIs may subvert physiological balance in the FRS. Preclinical studies confirm that anti-programmed cell death protein 1 (PD-1) therapy causes diminished ovarian reserve (40% reduction in primordial follicles) and ovulation failure in mice (6); clinical guidelines report menstrual disturbances or premature ovarian insufficiency in ~12% of premenopausal women receiving ICIs (7). This reproductive toxicity potentially stems from inflammatory cytokine storms, Treg cell depletion, and dysregulation of the estrogen-immune axis, creating a profound therapeutic paradox—anti-tumor immune activation risks functional impairment of reproduction (8).

Current reviews lack systematic analysis of this duality. This review integrates three pivotal dimensions: the immunological basis of FRS homeostasis and its vulnerability to therapeutic intervention; clinical efficacy and limitations of immunotherapy in gynecologic diseases; mechanistic insights into reproductive toxicity and strategies for fertility preservation. By synthesizing translational evidence from bench to bedside, we aim to guide safer clinical application of immunotherapies in women of reproductive age.

## 2 Overview of FRS diseases and immunotherapy

### 2.1 Immunological Characteristics of FRS diseases

The FRS’s immune system undertakes a unique dual function: defending against pathogen invasion while maintaining immune tolerance to“foreign”antigens such as sperm and embryos. Abnormal immune cell function at the maternal-fetal interface and immune dysregulation mediated by vaginal microbiota are associated with adverse pregnancy outcomes. At the maternal-fetal interface, dNK cells constitute the largest subset of lymphocytes, accounting for approximately 70% of endometrial leukocytes (9). They play a critical role in maintaining immune tolerance during pregnancy, protecting the mother from infection while avoiding immune rejection of the semi-allogeneic fetus (10). Ni Fang et al. integrated untargeted metabolomics and label-free quantitative proteomics to conduct paired analyses of dNK cells from four first-trimester deciduae and age-matched peripheral blood pNK cells. Using fluorescence-activated cell sorting, they validated reactive oxygen species (ROS) levels and found that dNK cells actively suppress cytotoxic pathways while upregulating adhesion/cytoskeletal remodeling proteins by downregulating glycerophospholipid and glutathione metabolism and elevating membrane-saturated lipids and intracellular ROS. This mechanism weakens their killing function and strengthens stable contact with trophoblasts, ultimately establishing a “redox-lipid metabolism-immune tolerance network” at the maternal-fetal interface to prevent embryo rejection by the maternal immune system (11). Immune cells such as T cells and macrophages (MΦ) are present in the female reproductive tract, regulating immune responses against invading pathogens and maintaining tissue homeostasis. Their dysregulation can lead to inflammation, impairing fertility. In aged mice, Treg cells exhibit increased interferon-γ (IFN-γ) secretion, whereas young Treg cells or aged Treg cells treated with α-PD-1 antibody elevate interleukin (IL)-10 levels, improving pregnancy outcomes. Additionally, IL-10-deficient mouse models show reduced frequencies of uterine CD4^+^CD25^+^Foxp3^+^ Treg cells, directly linked to disrupted maternal-fetal immune tolerance (12). In recurrent RSA patients, natural killer (NK) cells disrupt the HLA-G/TGF-b signaling pathway in human amniotic epithelial cells while exhibiting insufficient downregulation of cytotoxic molecules, leading to maternal-fetal immune tolerance imbalance (13). In RSA, reduced PD-1 protein expression on decidual MΦ cells and decreased Programmed Cell Death-Ligand 1(PD-L1) expression on placental villi disrupt the PD-1/PD-L1 axis, inducing M1 polarization (14). Knocking down CYP26A1 in the mouse uterus not only reduces embryo implantation but also significantly lowers TNF-α, IL-6, and CD86 protein levels, resulting in insufficient M1 polarization (15). Furthermore, in the immune profile of RSA without chromosomal abnormalities, the pro-inflammatory subset of CD11c-high MΦ cells is markedly increased (16). These findings suggest that MΦ cells in RSA patients may polarize toward a pro-inflammatory (M1) rather than an anti-inflammatory (M2) phenotype, triggering local inflammatory responses and placental dysfunction. Vaginal microbiota-mediated immune dysregulation at the maternal-fetal interface is associated with adverse pregnancy outcomes. In a prospective cohort study (n=152), Jiang et al. used longitudinal 16S rRNA sequencing to establish a causal link between first-trimester microbiota imbalance and subsequent immune instability. They found that a vaginal microbiota dominated by *L. iners* (CST III) in early pregnancy was strongly associated with an increased risk of recurrent spontaneous preterm birth, likely due to its weak antimicrobial capacity, poor microbiota stability, and tendency to shift toward non-lactobacillus-dominant (CST IVA/B) profiles. The latter induces local pro-inflammatory factors (e.g., CXCL10, MMP-9), compromising cervical barrier function and disrupting maternal-fetal immune balance, thereby triggering preterm birth (17). Chromosomally normal miscarriages are significantly linked to vaginal microbiota lacking lactobacilli, with elevated levels of IL-1β, IL-6, and IL-8 in cervicovaginal fluid compared to chromosomally abnormal miscarriages or healthy pregnancies (18).

The female reproductive tract epithelial cells and immune cells must simultaneously maintain tolerance to allogeneic sperm andsemi-allogeneic embryos while responding to a range of sexually transmitted pathogens, an immunological “dichotomy”unique to the FRS (19). At the same time, there are significant sex differences in the immune responses of the FRS, with sex hormones regulating reproductive functions and exerting diverse immunomodulatory effects on immune cells, including influencing T and B cell development, antibody production, lymphoid organ size, and lymphocyte death. For instance, progesterone and human chorionic gonadotropin affect T cell epigenetic and metabolic reprogramming by upregulating progesterone-induced blocking factor and promoting histone methylation, leading to an overall Th2 bias and increased Treg proportions, which negatively impact tumor immunity (20, 21). Estrogen enhances CD4^+^ T cell activation, particularly higher IL-15-mediated T cell activation observed in females, an effect likely mediated through estrogen receptor signaling pathways, though high estrogen concentrations may suppress certain immune activation differences (22). In female patients with papillary thyroid carcinoma and non-small cell lung cancer, larger immune cell aggregates are observed, resulting in more effective tumor immune surveillance and enhanced anti-tumor immunity, possibly attributed to more mature and diverse tumor-infiltrating B lymphocytes (Tertiary Lymphoid Structures, TLS) promoted by sex hormone pathways in females (23). In pancreatic adenocarcinoma, a significant positive correlation is observed between estrogen receptor 1 and estrogen receptor 2 levels and TLS scores in the TME, with female pancreatic adenocarcinoma (PAAD) patients exhibiting higher TLS scores and better prognosis, suggesting estrogen may play a key role in promoting TLS formation and enhancing anti-tumor immunity (24).

ICMs play a central role in maintaining maternal-fetal immune tolerance. Molecules such as PD-1 and its ligand PD-L1 may play a key role in the pathogenesis of pregnancy-specific diseases like preeclampsia by regulating maternal immune system function to sustain pregnancy. Research by Tripathi et al. found significantly reduced expression levels of PD-1 and PD-L1 in placental tissues of preeclampsia patients. The decrease in PD-L1 inhibits the expression of granulocyte-macrophage colony-stimulating factor by activating the JAK2/STAT5 signaling pathway, leading to diminished migration, invasion, and proliferation of trophoblast cells, as well as increased apoptosis. Additionally, in preeclampsia-like animal models, intravenous injection of PD-L1 overexpression vectors alleviated preeclampsia-like symptoms (e.g., hypertension, proteinuria), reduced placental, liver, and kidney damage, promoted fetal growth (increased fetal weight and length), and reversed the Treg/Th17 cell imbalance, improving fetal outcomes (25). The balance of ICMs such as B7-H1 and B7–1 is crucial for maintaining immune tolerance at the fetal-maternal interface, and any imbalance in the T helper 1 cell (Th1)/T helper 2 cell (Th2) immune system may lead to adverse pregnancy outcomes (26). Cross-analysis studies also identified B7-H4 as a “tumor-fetal” immune tolerance checkpoint, with its genetic deficiency causing immune activation and fetal resorption in allogeneic pregnancy models (27). T-cell immunoglobulin and mucin-domain containing-3 (Tim-3), as an inhibitory checkpoint protein, plays a key role in immune tolerance. Its abnormal expression in immune cells may be associated with RSA. Research data indicate that compared to normal pregnant women with Th1/Th2 imbalance, patients with unexplained recurrent pregnancy loss (RPL) exhibit significantly higher soluble Tim-3 expression (28). Recently, Cui et al. comprehensively delineated the core mechanistic role of the ZDHHC3-Tim-3-Cys9 palmitoylation-lysosomal degradation axis in decidual CD4^+^ T cell dysfunction through multidimensional data, including structural site mutations, protein stability tracking, lysosomal inhibitor interventions, and animal model validation. The absence of Tim-3 leads to significantly reduced secretion of immunosuppressive cytokines such as IL-10 and TGF-β by decidual CD4^+^ T cells, alongside elevated levels of IFN-γ and TNF-α, resulting in a Th1/Th17 shift at the maternal-fetal interface and ultimately triggering embryo resorption.

The impact of the IME on female reproductive processes has garnered increasing attention. In POI, the immune system plays a critical role, particularly with T lymphocytes being the most pivotal in the immunopathogenesis of autoimmune-related POI. Lymphocytic oophoritis, anti-ovarian autoantibodies, and concurrent autoimmune diseases are the main features of autoimmune POI (29). Recent studies have shown that POI patients exhibit enhanced Th1 responses and Treg cell deficiencies in both peripheral blood and ovarian tissues, leading to an elevated Th1/Treg ratio, which positively correlates with POI severity. Th1 cell-secreted IFN-γ and TNF-α synergistically promote granulosa cell apoptosis, inhibit steroidogenesis, and result in follicular atresia and ovarian failure (30). Approximately 30%-50% of POI patients have detectable anti-ovarian antibodies in their serum, targeting granulosa cell and oocyte antigens (e.g., POTEE, POTEF, etc.). These antibodies may exacerbate ovarian damage through immune complex deposition or complement activation (31, 32). A large-sample study by Wang et al., involving 610 cases with over 10 years of follow-up, first demonstrated at the population level that women with POI have a significantly and persistently elevated risk of autoimmune diseases, independent of family background (33).

Patients with endometriosis exhibit imbalances in immune cell subsets and the endocrine hormone-immune regulatory axis. These immunological alterations may be key factors or at least accomplices in impaired endometrial receptivity. For unexplained female infertility, studies have observed correlations between certain immune cell characteristics and infertility. High expression of HLA class II in adenomyotic endometrium can activate MΦ cells, stimulating T cells to secrete IL-6, IL-8, and IL-10. These cytokines stimulate B cells to produce immunoglobulins, triggering immune responses that may hinder embryo implantation. Abnormal cytokine secretion by endometrial MΦ cells and immune cell interactions could create an immunological “vicious cycle,” exacerbating adenomyosis-related infertility (34). Subgroup analysis of an adenomyosis cohort showed no increase in uNK cell numbers in women with mild adenomyosis, whereas elevated uNK cell counts were observed in the normal endometrium of women with severe adenomyosis during the late luteal phase (cycle days 22–26). However, increased uNK cell concentration in adenomyosis may also affect endometrial receptivity, leading to infertility and miscarriage, which requires further confirmation (35–38). Other findings revealed increased CD8^+^ T cells and CD56^+^ NK cells alongside decreased CD163^+^ MΦ cells in the endometrium of endometriosis patients, highlighting a pro-inflammatory signature in the endometrial immune environment. Elevated CD8^+^ T cells were associated with higher infertility risk in affected women (39). Wang et al. analyzed a retrospective cohort of 317 women with reproductive disorders (117 with a surgical history of endometriosis, 200 without) and confirmed that regardless of lesion location, a history of endometriosis surgery independently correlated with reduced peripheral NK cell cytotoxicity and increased endometrial CD68^+^ MΦ cell infiltration. Pearson correlation analysis showed a relationship between serum E_2_ levels and endometrial CD68^+^ MΦ cell proportion. Further integration with the EndometDB public database revealed mild positive correlation between CD68 expression and estrogen receptor β, suggesting the E_2_–estrogen receptor β2 axis may recruit MΦ cells via upregulated chemokines like CCL2, contributing to endometriotic lesion microenvironment formation (40). Single-cell transcriptome studies identified unique immune cell cluster distributions in peripheral blood mononuclear cells of patients with ovulatory dysfunction (e.g., POI and menopause), showing differences in 7 major cell types and 25 subpopulations compared to healthy individuals, indicating that IME disturbances may contribute to infertility by affecting follicular development and ovulation (41).

### 2.2 Current applications of immunotherapy in FRS diseases

Gynecological malignancies (including ovarian cancer, cervical cancer, and endometrial cancer) pose a significant threat to women’s health worldwide. Although traditional treatments such as surgery, chemotherapy, and radiotherapy are widely used, they face limitations in efficacy, significant side effects, and drug resistance (42, 43). In recent years, immunotherapy has emerged as a novel strategy, demonstrating potential in some gynecological cancers by activating the body’s immune system to combat tumors. Currently, ICIs and CAR-T therapy are research hotspots, but their overall application remains exploratory, particularly facing numerous challenges in the treatment of solid tumors (44–46).

ICIs (such as anti-PD-1/PD-L1 antibodies) have demonstrated significant efficacy in certain gynecologic malignancies, particularly in advanced or recurrent cases (47). For instance, ICIs have been approved by the U.S. Food and Drug Administration for the treatment of cervical and endometrial cancers, providing durable responses for some patients. In a single-arm phase II clinical trial (NCT03241745), the efficacy and safety of the PD-1 inhibitor nivolumab were evaluated in 35 patients with dMMR or high tumor mutational burden uterine or ovarian cancer. The primary endpoints were objective response rate (ORR) and 24-week progression-free survival (PFS). Researchers also found that interactions between CD8^+^PD-1^+^ or CD8^+^PD-1^+^TOX^+^ T cells and PD-L1+ cells, as well as somatic mutations in MEGF8 or SETD1B, were associated with PFS, suggesting potential biomarkers for PD-1 blockade therapy (48). In a multi-arm, open-label phase I/II study, 40 patients with heavily pretreated advanced gynecologic cancers were enrolled into six treatment arms. The data showed limited overall responses, but specific subgroups (e.g., cervical cancer) may achieve sustained disease control with the combination of avelumab and a 4-1BB agonist. Anti-PD-1 combined with anti-cytotoxic T-lymphocyte-associated protein 4 (CTLA-4) has also shown notable clinical benefits in rare gynecologic cancers (49). However, Kuang W et al. conducted a retrospective analysis of 1,333 gynecologic cancer patients at West China Second University Hospital of Sichuan University from 2021 to 2024, revealing no statistically significant differences in median PFS or median overall survival (OS) between MSI-H and MSS endometrial cancer patients. Integrating multi-omics data from TCGA (n=10,437) and MSK-IMPACT (n=28,963) totaling 39,400 tumors, researchers found that lowering the high TMB threshold to 6–8 mutations per megabase (mut/Mb) could increase the proportion of Black and Asian patients eligible for ICIs without compromising predictive efficacy. Among 1,594 patients with objective response data who received ICI therapy, a TMB ≥6 mut/Mb was significantly associated with response rates in White and Black patients. Asian patients also showed a trend toward benefit at ≥8 mut/Mb, suggesting this threshold retains predictive value for ICI benefit across ethnicities (50). In the GARNET phase I trial, dostarlimab monotherapy demonstrated durable antitumor activity in mismatch repair-deficient (dMMR/MSI-H) endometrial cancer patients, achieving an ORR of 42.3% in dMMR patients and a median duration of response of 34.7 months (51). Thus, ICIs remain limited in microsatellite-stable or mismatch repair-proficient tumors, highlighting the need for further exploration of biomarkers and combination strategies to expand the beneficiary population. CAR-T cell therapy has achieved breakthrough success in hematologic malignancies, and CAR-T therapies targeting specific antigens in gynecologic cancers have shown antitumor effects in preclinical studies. Mesothelin-targeted CAR-T cells with a CD28 costimulatory domain (M28z) significantly prolonged survival in ovarian cancer mouse models but failed to achieve durable tumor control. Compared to CAR-T cells with a 4-1BB costimulatory domain (MBBz), M28z exhibited stronger cytolytic activity and cytokine release capacity (52, 53). NKG2D CAR-T cells demonstrated significant killing effects against ovarian cancer cell lines *in vitro* and *in vivo*, with no observed off-target toxicity. NKG2D CAR-T cells also significantly suppressed cervical tumor growth without apparent toxicity, suggesting their potential as a novel cellular therapy (54). Preliminary clinical studies indicate that CAR-T therapy has some efficacy in certain gynecologic cancer patients, but its application is limited by severe adverse effects, such as cytokine release syndrome (CRS) and graft-versus-host disease (GVHD) (43).

## 3 Clinical impact of immunotherapy on the FRS

### 3.1 Application of ICIs and related clinical studies

ICIs may negatively affect female reproductive system function. Studies indicate that ICIs can increase immune cell infiltration in the ovaries and tumor necrosis factor-α expression, reduce ovarian follicle reserves, and impair oocyte maturation and ovulation capacity. Data suggest that ICIs may harm both current fertility and future reproductive potential in women. Therefore, fertility preservation measures are strongly recommended for female patients undergoing ICI therapy (55). ICIs may trigger various irAEs, including skin toxicity, thyroid dysfunction, pancreatitis, cholangitis, and rheumatologic disorders (56, 57). Clinical observations also reveal that patients receiving immunotherapy often experience sexual dysfunction, decreased libido, and reduced vaginal lubrication (58). These adverse effects can impact multiple organ systems and may even lead to disability or death (59). Glucocorticoids are the first-line treatment for irAEs, but second-line therapies must be explored for steroid-refractory cases (60). Additionally, the potential impact of ICIs on the FRS warrants special attention, and fertility preservation measures should be prioritized in clinical management.

The therapeutic efficacy of ICIs in gynecologic tumors has demonstrated significant clinical benefits, particularly in advanced or recurrent cases as previously mentioned. ICIs have been approved by the U.S. Food and Drug Administration for the treatment of cervical and endometrial cancers, providing durable therapeutic effects for some patients (61). Additionally, ICIs have shown certain efficacy in rare histological subtypes such as ovarian and vulvar cancers (47). However, resistance to ICIs remains a major challenge in the treatment of some gynecologic malignancies, especially in ovarian cancer, where their efficacy remains suboptimal (47). Furthermore, factors such as the complexity of the TME, the presence of immunosuppressive mechanisms, and significant interpatient heterogeneity severely limit the effectiveness of immunotherapy (62, 63). The TME cannot be simply categorized as a purely “anti-tumor” or “pro-tumor” environment but is rather a dynamic and plastic system characterized by hypoxia, nutrient deprivation, inflammation, immunosuppression, and angiogenesis. In gynecologic tumors (e.g., ovarian and cervical cancers), the TME contains various immunosuppressive cell populations, including tumor-associated macrophages (TAMs), myeloid-derived suppressor cells (MDSCs), and regulatory T cells (Tregs). These cells form an immunosuppressive network by secreting cytokines such as IL-10 and TGF-β, impairing the cytotoxic function of effector T cells (64). Tumor cells induce changes in surrounding stromal cells, such as the transformation of fibroblasts into cancer-associated fibroblasts (CAFs). CAFs directly promote tumor cell proliferation, migration, and invasion by secreting various growth factors (e.g., IGF1, ELN), cytokines, and chemokines (65). Meanwhile, Tregs and MDSCs establish a mutually reinforcing immunosuppressive network within the TME. MDSCs not only directly suppress T-cell function but also promote Treg expansion, while Tregs further enhance the immunosuppressive activity of MDSCs by secreting cytokines like IL-10 and TGF-β. This positive feedback loop leads to progressively intensified immunosuppression in the TME (66).

Research on immunotherapy for early-stage malignant tumors is relatively scarce, and there is insufficient data on the long-term efficacy and safety of immunotherapy, particularly regarding the optimal strategies (such as timing, dosage, and sequence) when combined with other treatment modalities (e.g., surgery, chemotherapy) (67, 68). Exploring combination therapy strategies is a crucial direction for improving the efficacy of ICIs. The combination of ICIs with angiogenesis inhibitors or chemotherapy drugs has shown potential in clinical trials. KEYNOTE-146 and KEYNOTE-775 demonstrated that combining anti-PD-1 ICIs with vascular endothelial growth factor inhibitors prolonged survival in patients with platinum-refractory endometrial cancer. Currently, the phase III randomized LEAP-001 trial (NCT03884101) is evaluating this combination therapy as first-line treatment for stage III, IV, or recurrent endometrial cancer (69). ICIs are also being combined with poly(ADP-ribose) polymerase (PARP) inhibitors as dual therapy (NCT03016338) or with chemotherapy (DUO-E trial) and anti-vascular endothelial growth factor therapy (EndoBARR trial) as triple therapy. The KEYNOTE-426 study, conducted in 86 previously untreated patients with advanced clear cell renal cell carcinoma, further established this combination regimen as the first-line standard treatment for advanced renal cancer (69). Additionally, the combination of ICIs with DNA damage repair inhibitors or PARP inhibitors is under investigation to overcome resistance and enhance therapeutic efficacy. A recent phase I/II clinical study (NCT02657889) preliminarily demonstrated the putative synergistic effect of this combination, showing that niraparib/pembrolizumab combination therapy achieved considerable therapeutic outcomes in patients with refractory ovarian cancer, significantly surpassing niraparib or pembrolizumab as monotherapy (70). Another clinical study for recurrent ovarian cancer patients (NCT02484404) moderately validated the putative therapeutic combination and further suggested the inhibition of vascular endothelial growth factor (VEGF) and vascular endothelial growth factor receptor (VEGFR) as potential synergistic factors (71). However, combination therapy may also increase the risk of irAEs, necessitating further optimization of treatment regimens (72). The development and application of biomarkers are critical for predicting the efficacy and adverse effects of ICIs. dMMR and MSI-H, as core predictive biomarkers for ICI efficacy, have formed a systematic evidence chain in gynecologic malignancies, with their value validated through multiple clinical trials. The KEYNOTE-158 trial showed that 90 patients with dMMR/MSI-H advanced endometrial cancer treated with pembrolizumab directly led the FDA to incorporate dMMR/MSI-H testing into companion diagnostic standards (73). The CheckMate142 trial further confirmed that the concordance between local and central dMMR/MSI-H testing reached 72%, with the ORR in centrally confirmed positive patients significantly higher than in unconfirmed cases, highlighting the importance of standardized testing for identifying beneficiary populations (74). Epidemiologically, a multicenter study by Park et al. involving 1,093 gynecologic cancer patients found the highest incidence of dMMR/MSI-H in endometrial cancer, with dMMR/MSI-H patients showing significantly higher ORR to ICIs than mismatch repair-proficient patients, confirming its cross-cancer predictive value (75). The 2024 American Cancer Society guidelines explicitly recommend pembrolizumab monotherapy or combination chemotherapy as a preferred second-line regimen for dMMR/MSI-H advanced cervical cancer patients, with efficacy not significantly associated with PD-L1 expression (76). At the mechanistic level, studies in endometrial cancer reveal that dMMR/MSI-H tumors generate abundant neoantigens due to high mutational burden, accompanied by increased CD8+ T-cell infiltration and upregulated PD-L1 expression, creating an IME favorable for ICI efficacy (76). However, in ovarian cancer, the overall incidence of MMRd/MSI-H is low, and preliminary data indicate a lower ORR in MSI-H ovarian cancer patients treated with ICIs, suggesting the need for larger sample sizes to further validate MSI-H as a predictive biomarker (75). Unlike the relative stability of dMMR/MSI-H, PD-L1 immunohistochemical expression exhibits significant heterogeneity in gynecologic malignancies. In cervical cancer, PD-L1-positive (CPS ≥1) patients had significantly higher ORR and prolonged median overall survival to 11.26 months (77). However, this predictive value varies by molecular subtype in endometrial cancer. A systematic review by De Tommasi et al., encompassing 39 studies (3,844 endometrial cancer cases), found that PD-L1 (CPS ≥1) positivity was higher in POLE ultramutated tumors than in MSI-H tumors. Additionally, analysis of 12 paired samples revealed significant discordance in PD-L1 expression between primary and metastatic lesions, emphasizing the need for individualized assessment based on tumor stage and molecular subtype (78). Current research has confirmed that predictive models integrating multiple biomarkers significantly improve accuracy. For example, in cervical cancer, models incorporating plasma proteins such as TGF-α markedly enhanced predictive performance (79). In a 2024 multicenter study, Grau Bejar et al. used spatial multiplex immunofluorescence to analyze 94 MMRd/MSI-H endometrial cancer cases and found that integrating ieCD8^+^, stromal CD8^+^, PD-L1, and HLA-I into a four-parameter “immune score” significantly improved the ROC for ICI response prediction compared to MMRd status alone. Concurrently, TGF-β pathway activation and high expression of the hypoxia-related marker S100A2 were strongly associated with acquired resistance, suggesting the need for personalized evaluation based on molecular subtypes and the TME (80). In summary, dMMR/MSI-H, with its clear predictive value and standardized testing methods, has become a core biomarker for ICI therapy in gynecologic cancers. Meanwhile, dynamic PD-L1 assessment and multidimensional biomarker integration provide more precise foundations for individualized treatment decisions, advancing immunotherapy from single-marker guidance to multi-omics precision medicine.

### 3.2 The dual role mechanism of CD8^+^HLA-DR^+^ cells in cancer immunotherapy and pregnancy miscarriage

In studies on the impact of immunotherapy on the FRS, CD8^+^ HLA-DR^+^ cells, as a highly activated Tcell subset, are increasingly recognized as a critical bridge linking antitumor immune responses with pregnancy immune tolerance. The functional state differences exhibited by this cell subset in various immune environments may profoundly influence cancer treatment efficacy and pregnancy outcomes. To gain deeper insights into its mechanistic roles in these two pathological states, analyses must be conducted separately from the dimensions of cancer immune activation and miscarriage-related immune imbalance.

In the context of cancer, CD8^+^HLA-DR^+^ cells are generally regarded as markers of immune system activation. Studies indicate that these cells often co-express activation or inhibitory molecules such as CD38, PD-1, T-cell immunoglobulin, and ITIM domain proteins, displaying typical inflammatory response characteristics. Notably, an increased frequency of CD8^+^HLA-DR^+^CD38high T cells has been shown to correlate with better responses to anti-PD-1 therapy and longer progression-free survival in non-small cell lung cancer patients, suggesting this cell subset may serve as a predictive biomarker for ICIs efficacy (81). However, excessive immune system activation can also lead to T-cell exhaustion. For instance, persistent antigen stimulation may drive CD8^+^HLA-DR^+^ cells to adopt an “exhausted” phenotype, such as CD38^+^PD-1^+^, ultimately impairing their cytotoxic function (82). Furthermore, the role of CD8^+^HLA-DR^+^ cells in tumor immunotherapy resistance is gradually being uncovered. In some cancer patients, these cells not only express exhaustion markers but also exhibit metabolic dysregulation—for example, CD38-mediated metabolic reprogramming (e.g., cholesterol metabolism) can weaken CD8^+^ T-cell function and promote immunotherapy resistance (83). Additionally, the tumor-associated IME significantly modulates the state of CD8^+^ HLA-DR^+^ cells. Monocytes facilitate their recruitment via the CXCL16–CXCR6 axis, while Treg cells suppress their activity through the CCR7/CCL19 pathway, forming a complex intercellular interaction network that collectively shapes the quality and intensity of antitumor immune responses (83).

In contrast to the enhanced T-cell activity required for tumor immune system activation, the pregnancy process heavily relies on a locally immunosuppressive environment, particularly in the decidua and placental regions. Therefore, abnormal activation of CD8^+^HLA-DR^+^ cells during pregnancy may adversely affect the embryo. In normal pregnancies, the decidua is enriched with immunosuppressive CCR8^+^Treg cells, which are crucial for maintaining maternal-fetal immune tolerance. Their reduction is closely associated with RPL (84). In RPL patients, the proportion of CD8^+^HLA-DR^+^ cells in peripheral blood is significantly elevated alongside downregulated PD-1 expression, suggesting that their hyperactivation may disrupt the existing immune tolerance mechanisms, leading to pregnancy failure (85). Concurrently, CD8^+^HLA-DR^+^ cells collaborate with decidual NK cells in the immune mechanisms of miscarriage. Studies show that in RPL patients, reduced CCL1 production by decidual CD49a^+^NK cells leads to insufficient recruitment of CCR8^+^Treg cells, resulting in uncontrolled expansion of CD8^+^T cells and escalated local inflammation. The simultaneous elevation of CD8^+^HLA-DR^+^ cells and CD56^dim^CD57^+^NK cells in peripheral blood may form an inflammatory axis triggering immune-mediated miscarriage. More critically, dysregulated immune checkpoint signaling may provide a common explanatory framework for these two pathological states (86). PD-L1, a key factor in maintaining maternal-fetal immune tolerance, exhibits downregulated expression in the placenta or reduced soluble PD-L1 levels in maternal serum, both of which can release CD8^+^HLA-DR^+^ cells from inhibition, leading to trophoblast cell attack and placental damage (87). Animal experiments further validate this mechanism: adoptive transfer of CCR8^+^Treg cells effectively rescues pregnancy failure caused by immune imbalance, demonstrating that abnormal activation of CD8^+^HLA-DR^+^ cells can be reversed by immunomodulatory strategies (84).

Although cancer and miscarriage appear to represent two extremes—”immune enhancement” and “immune tolerance deficiency,” respectively—they are both fundamentally linked to PD-1/PD-L1 axis imbalance. Tumors evade immune surveillance by mimicking the maternal-fetal immune tolerance environment (e.g., high PD-L1 expression), while pregnancy failure stems from dysfunction of this signaling axis. This mechanistic overlap suggests that precise modulation of CD8^+^HLA-DR^+^ cells (via strategies targeting CD38, CCR8, or enhancing the PD-L1 pathway) may serve as a novel therapeutic target for both conditions, achieving a balance between sustaining pregnancy and controlling tumors. For instance, vitamin D adjuvant therapy in RPL patients can upregulate Treg cells to indirectly suppress CD8^+^HLA-DR^+^ cells (88). In summary, CD8^+^HLA-DR^+^ cells play dual roles in cancer immunotherapy and pregnancy failure, mediating immune effector enhancement and tolerance dysregulation, respectively. Their state is shaped by intracellular metabolism, phenotypic marker expression, and the local immune microenvironment. A deeper understanding of their functional network not only aids in predicting cancer immunotherapy outcomes but also provides critical immune intervention insights for preserving fertility in women of reproductive age.

### 3.3 Exploration of CAR-T Cell therapy in the FRS

CAR-T cell therapy, as a cutting-edge technology in immunotherapy, has demonstrated potential value in treating FRS diseases, particularly gynecological tumors, while also facing numerous challenges. Progress has been made in target research for ovarian cancer, with CAR-T therapies targeting mesothelin, MUC16, and FOLR1 showing significant antitumor activity in preclinical models, effectively inhibiting tumor cell growth (89, 90). Additionally, EGFR-targeted CAR-T cells exhibited notable cytotoxic effects against EGFR-positive gynecological malignancies such as ovarian and endometrial cancers *in vitro*. Compared to conventional EGFR-CAR-T, modified versions—EGFR-DNR-CAR-T (dominant-negative TGF-β receptor 2) and EGFR-SMAD7-CAR-T (overexpressing SMAD7)—demonstrated stronger proliferation and tumor-lytic capabilities against EGFR-positive tumor cells (e.g., A549) in vitro. TGF-β-neutralizing antibodies further enhanced the activity of conventional EGFR-CAR-T. These modifications, by counteracting the immunosuppressive TGF-β signaling in the TME (SMAD7 is a negative regulator of TGF-β downstream signaling), significantly improved the antitumor efficacy of CAR-T cells (91, 92). Fibroblast activation protein (FAP) has emerged as a potential target, with theoretical foundations and preclinical studies of its CAR-T therapy gradually expanding in solid tumors, including gynecological malignancies (93). Although CAR-T therapy has achieved remarkable success in hematologic malignancies, clinical data in gynecological solid tumors remain relatively scarce. While some early clinical trials have shown promise, overall efficacy requires further validation. For example, MSLN-targeted CAR-T cells demonstrated antitumor effects in ovarian cancer xenograft models, but substantial optimization is still needed for successful clinical translation (90).

Gynecological tumors such as ovarian cancer contain abundant immunosuppressive cells (e.g., MDSCs) and Tregs in their microenvironment, which directly suppress the activity and proliferation of CAR-T cells by secreting inhibitory cytokines like TGF-β and IL-10. Studies show that the persistence of CAR-T cells (e.g., M28z and MBBz-modified CAR-Ts) in ovarian cancer models is significantly limited by co-inhibitory pathways (e.g., PD-1/PD-L1), leading to increased exhaustion phenotypes (94). High concentrations of adenosine and lactate (produced via glycolysis) in the TME inhibit CAR-T cell function through mechanisms such as A2A receptor activation—adenosine reduces the cytotoxicity of human CAR-T cells, while lactate promotes the polarization of immunosuppressive MΦ cells (94). The dense stroma and abnormal vascular structure of ovarian cancer hinder CAR-T cell infiltration into tumor sites. CAFs secrete extracellular matrix (ECM) components (e.g., collagen), forming a physical barrier that restricts CAR-T cell migration. Experiments indicate that after migrating from blood vessels into the stroma, CAR-T cells may become trapped in the ECM and fail to reach tumor cells (95). In high-grade serous ovarian cancer, distinct CAF subsets (e.g., COL11A1-high subsets) contribute to barrier formation by secreting specific ECM components (e.g., collagen XIα1). Matched patient sample analyses reveal dynamic changes in ECM secretion patterns of CAF subsets in post-chemotherapy metastases, further exacerbating CAR-T cell infiltration barriers (96).

Toxicity issues cannot be overlooked, as side effects such as CRS and GVHD significantly limit the widespread clinical application of CAR-T therapy (43, 97). Most CAR-T therapy-related studies remain in the preclinical or early trial stages, lacking large-scale Phase III clinical trial data has demonstrated potential value in treating FRS diseases, Utilizing CRISPR gene-editing technology holds promise for enhancing the persistence and safety of CAR-T cells or developing “off-the-shelf” CAR-T cells, reducing the cost and complexity of personalized treatment (43, 98, 99). Combining CAR-T therapy with ICIs (e.g., PD-1/PD-L1 inhibitors) or chemotherapy may help reverse the immunosuppressive TME and improve therapeutic efficacy (100, 101). CAR-NK cell therapy, with its safety advantages and “off-the-shelf” potential, demonstrates broader application prospects in gynecologic tumor treatment compared to CAR-T therapy (43).

### 3.4 Research needs on potential effects on reproductive function

Although the infertility risks of traditional chemotherapeutic agents (such as alkylating agents and platinum-based drugs) are well-established, research on the reproductive toxicity of novel immunotherapeutic agents remains in its early stages. Preclinical animal models indicate that various emerging therapies may adversely affect reproductive capacity, necessitating urgent attention (102, 103). The application of immunotherapy in patients of reproductive age requires careful consideration. Current evidence suggests that such treatments may impair reproductive potential, warranting comprehensive evaluation from mechanisms of action to preclinical and clinical trials (102, 104). While immunotherapy and other targeted therapies in gynecologic malignancies show promise in improving efficacy and reducing toxic treatments or surgeries with significant negative impacts on fertility, their potential risks to reproductive function still require further investigation (105). Currently, clinical evidence on the potential gonadotoxicity of targeted drugs and immunotherapy is very limited, and their specific effects on reproductive function in different patient populations (e.g., melanoma, lung cancer, breast cancer patients) urgently need clarification (102, 106). Additionally, in-depth study of the phenotype and function of uterine NK cell subsets may provide potential diagnostic and therapeutic targets for women with unexplained reproductive dysfunction (107). Future efforts should also increase support for translational research on reproductive immunology mechanisms to fill knowledge gaps in this field (108).

## 4 Basic mechanism research of immunotherapy on the female reproductive system

### 4.1 Molecular targets and pathways related to immunotherapy

The core of immunotherapy lies in enhancing anti-tumor immune responses by regulating key molecular targets and signaling pathways of the immune system. Currently, the most extensively studied molecular targets mainly fall into four categories: PD-1/PD-L1 and cytotoxic T-lymphocyte-associated protein 4 (CTLA-4) among ICMs are the most classic targets. These molecules maintain immune tolerance by inhibiting T-cell function, and inhibitors targeting them can relieve T-cell suppression and enhance anti-tumor immunity (109, 110). A double-blind randomized controlled trial systematically compared the clinical efficacy and safety of IBI310 combined with sintilimab versus placebo combined with sintilimab in recurrent/metastatic cervical cancer. The results showed that the IBI310-sintilimab group demonstrated significant advantages in the aforementioned metrics, including markedly prolonged progression-free survival and improved objective response rates. Additionally, safety analysis systematically evaluated adverse events in both groups, confirming the safety profile of this combination therapy (111). Olson et al. reported that for advanced melanoma patients who failed anti-PD-1/L1 therapy, pembrolizumab combined with low-dose ipilimumab achieved a balance between efficacy and safety (112). Du Y et al. developed a highly stable bifunctional aptamer that simultaneously blocks PD-L1 and CD47, synergistically enhancing the anti-tumor activity of T cells and macrophages (MΦ cells), significantly inhibiting hepatocellular carcinoma growth and prolonging survival in tumor-bearing mice with a favorable safety profile (113). PD-1/CTLA-4 combination therapy has shown clinical activity in some rare gynecological tumors, particularly in chemotherapy-resistant patients (49). The mechanisms of ICMs primarily regulate immune responses as follows: CTLA-4 acts during the early stages of T-cell activation by competitively binding B7 molecules (CD80/CD86) with CD28, inhibiting T-cell co-stimulatory signals and thus suppressing T-cell activation (109, 114). In the tumor microenvironment(TME), PD-1 binding to its ligand PD-L1 inhibits T-cell receptor signaling and metabolic reprogramming (e.g., suppressing glycolysis), leading to T-cell exhaustion (110, 115). These checkpoint molecules collaboratively maintain immune tolerance through distinct mechanisms, and their blockade can restore T-cell function and enhance anti-tumor immune responses (110, 116). While immunotherapy-induced inflammation can effectively combat tumors, it may also cause harmful side effects on healthy tissues, including reproductive organs. For instance, inflammation can alter the ovarian and uterine microenvironments. Uterine inflammation is associated with reduced embryo implantation, while ovarian inflammation may accelerate ovarian aging and diminish ovarian reserve, which is crucial for long-term fertility (117). Studies indicate that treatment with anti-mouse PD-1 antibodies upregulates pro-inflammatory cytokines TNF-α and cyclooxygenase-2 (COX-2), an enzyme primarily responsible for inflammation (118). Metabolic pathway-related targets play a critical role in immune cell metabolic reprogramming, and their imbalance may contribute to immunotherapy-related inflammatory responses and reproductive toxicity. The activation, differentiation, and effector functions of immune cells depend on metabolic reprogramming, a process particularly vital in the TME. Tumor cells and immune cells (e.g., T cells, NK cells) compete for limited nutrients (e.g., glucose, amino acids), impairing immune cell function. In gynecological tumors such as ovarian cancer, this metabolic competition may exacerbate the immunosuppressive microenvironment, thereby weakening immunotherapy efficacy (119). Cell death pathways, such as ferroptosis, exhibit cross-regulation with inflammatory responses, influencing immunotherapy outcomes through iron metabolism and lipid peroxidation pathways. For example, the ferroptosis inducer RSL3 increases tumor cell lipid peroxidation and immunogenicity, promoting dendritic cell activation and T-cell infiltration, thereby improving the TME (120). However, ferroptosis may also impair immune cell function. Iron overload and lipid peroxidation in MΦ cells or T cells can weaken their anti-tumor activity, leading to immune surveillance failure. This dual effect highlights the need for precise regulation of iron metabolism to avoid immune cell damage (121).

The mechanisms of inflammation induced by immunotherapy and its effects on the reproductive system are mainly reflected in two aspects: Mechanisms of inflammation:(1) Cytokine release: ICIs can increase levels of pro-inflammatory cytokines (e.g., TNF-α, IL-6, IL-1β), triggering systemic inflammatory responses (122–124). (2) Metabolic dysregulation: Dysfunction of the AMPK/mTOR pathway may lead to excessive immune cell activation, exacerbating inflammation (125–127). (3) Oxidative stress: Immunotherapy may increase ROS production by activating pathways such as nicotinamide adenine dinucleotide phosphate oxidase, further promoting inflammation (128). Effects on the reproductive system: (1) Gonadal function: Immunotherapy may cause gonadal damage through inflammatory responses, manifesting as sexual dysfunction, decreased libido, and abnormal hormone levels (58, 104). (2) Ovarian function: ICIs may lead to premature ovarian failure and infertility by disrupting the follicular microenvironment or inducing ovarian inflammation (55, 129). (3) Fertility preservation: Long-term data on the impact of immunotherapy on fertility are lacking, but animal studies suggest certain immunotherapeutic drugs may affect pregnancy maintenance by interfering with Treg cell function. For example, in a mouse model prone to miscarriage, defective Treg function due to abnormal IL-27/Blimp-1 axis can be improved by adoptive transfer of Tregs Blimp-1+ cells, reducing embryo resorption rates. This indicates that Treg-targeted immunomodulation strategies may influence pregnancy outcomes (130).

### 4.2 Potential effects of immunotherapy on normal reproductive function

Immunotherapy (particularly ICIs) has become a crucial treatment for various cancers, but its potential impact on the reproductive system remains unclear. Current research suggests that immunotherapy may affect reproductive function through the following pathways: gonadotoxicity (e.g., ICIs may directly act on gonadal tissues, leading to impaired spermatogenesis in males (such as oligospermia or azoospermia) or decreased ovarian reserve in females), endocrine disruption (e.g., by interfering with hormone secretion in the hypothalamic-pituitary-gonadal (HPG) axis, disrupting reproductive hormone balance and causing menstrual disorders or testosterone suppression), and inflammatory responses (e.g., related cytokine storms (e.g., elevated IL-6, TNF-α) may further activate inflammation via pathways like AMPK/mTOR, damaging the ovarian microenvironment or seminiferous tubule function (55, 131–134). The direct effects on ovarian function are particularly notable: (1) Decreased ovarian reserve: Clinical data indicate that immunotherapy may accelerate follicular atresia, reduce the primordial follicle pool, and significantly lower ovarian reserve (55). For instance, reduced follicle counts and ovulation disorders were observed in mouse models treated with anti-PD-1 therapy (55). In patients with inflammatory bowel disease, immunomodulators (e.g., thalidomide) have been associated with diminished ovarian reserve (135), suggesting that immunomodulation may directly impair ovarian function. (2) Oocyte quality and ovulation disorders: ICIs may disrupt mitochondrial function in granulosa cells or induce oxidative stress (e.g., ferroptosis triggered by iron overload), affecting oocyte maturation and ovulation (136). In animal models, excessive T-cell activation due to immunotherapy may attack ovarian tissue, triggering autoimmune oophoritis and further impairing ovulation (55, 137). (3) Hormonal abnormalities: Impaired granulosa cell function may lead to disrupted estrogen and progesterone secretion, affecting endometrial receptivity and embryo implantation. For example, thyroid dysfunction (associated with immunotherapy) can interfere with ovarian hormone secretion via the HPG axis (133).

Current research demands on the mechanisms of reproductive dysfunction are urgent: (1) Molecular mechanisms of immunotherapy and gonadal toxicity: Clarify the physiological roles of immune checkpoints (e.g., CTLA-4, PD-1) in the ovaries and testes and how their inhibition triggers gonadal cell apoptosis or ferroptosis (136, 138); Investigate the role of immune cells (e.g., Treg cells, NK cells) in maintaining ovarian immune tolerance and how immunotherapy disrupts this balance (131, 137); (2) Fertility preservation strategies: Explore whether antioxidants (e.g., coenzyme Q10) or mitochondrial protectants (e.g., melatonin) can alleviate immunotherapy-induced ovarian oxidative damage (136, 139); Evaluate the applicability of fertility preservation techniques (e.g., oocyte cryopreservation or ovarian tissue transplantation) in immunotherapy patients (55, 129); (3) Lack of clinical data: Most current evidence comes from animal models or small-scale retrospective studies, highlighting the need for prospective clinical studies to assess the long-term effects of immunotherapy on female anti-Müllerian hormone levels and male semen parameters (58, 102, 140), and to establish standardized monitoring protocols, such as dynamic evaluation of ovarian function through serum hormone levels (134, 141); (4) Sex differences and personalized treatment: Immunotherapy may differentially impact male and female reproductive function (e.g., females are more prone to premature ovarian failure, while males primarily exhibit spermatogenic dysfunction) (131, 132), and targeted studies should incorporate patient genetic backgrounds to analyze individual differences in immunotherapy risks (142).

### 4.3 The Role of the IME in FRS diseases

The IME of the FRS plays a crucial role in maintaining reproductive health and fertility. Through the coordinated actions of various immune cells (such as MΦ cells, dendritic cells, NK cells, and B cells), the IME regulates immune responses to pathogens and maintains tissue homeostasis. For instance, immune cells in the endometrium modulate endometrial receptivity via intercellular communication during the implantation window, ensuring successful embryo implantation (143, 144). Additionally, immune tolerance mechanisms (e.g., the role of Treg cells) are essential for preventing maternal immune attacks on the embryo, particularly in early pregnancy (137). The ovarian microenvironment supports reproductive function by regulating follicular development and oocyte maturation, where interactions between immune cells and stromal cells are indispensable for maintaining ovarian function (145, 146). However, dysregulation of the IME can lead to various reproductive disorders. For example, chronic low-grade inflammation may induce ovarian oxidative stress and tissue fibrosis, contributing to conditions such as polycystic ovary syndrome, endometriosis, and ovarian aging (147). In patients with recurrent implantation failure, an imbalance in the Th1/Th2 cell ratio and an increase in pro-inflammatory monocyte-derived MΦ cells can remodel the ovarian IME, accelerating reproductive decline (144, 145). Furthermore, disturbances in the endometrial IME (e.g., chronic endometritis) can impair endometrial receptivity, leading to infertility or assisted reproductive technology failure (148).

Immunotherapy (such as ICIs) fights diseases by activating the immune system, but its intervention in the IME of the FRS may have dual effects. On one hand, immunotherapy can remodel the TME, for example, by enhancing antitumor effects through combined therapy targeting ferroptosis and immune checkpoint blockade (149, 150); in human papillomaviruspositive head and neck squamous cell carcinoma, research on the IME provides new directions for personalized treatment (151). On the other hand, excessive activation of immunotherapy may disrupt immune tolerance in the reproductive system, triggering irAEs. For instance, ICIs may cause reproductive system side effects, such as ovarian function impairment or infertility, due to systemic immune overactivation (55, 152). Clinical data show an association between ICI treatment and FRS disorders (such as hormonal imbalances and increased risk of miscarriage) (152, 153). Additionally, certain drugs (such as triptolide) may have immunomodulatory effects but could also exert toxicity on the FRS, including damage to ovarian and endometrial function (154). Similarly, environmental toxins (such as polychlorinated biphenyls and perfluoroalkyl substances) can interfere with the IME, leading to reduced pregnancy rates, hormonal imbalances, and reproductive diseases (153, 155). In recurrent ovarian cancer, the heterogeneity of the IME necessitates personalized treatment strategies to balance efficacy and side effects (156).

### 4.4 Abnormal changes in immune indicators during immunotherapy and their association with reproductive failure

While activating anti-tumor immunity, immunotherapy may disrupt maternal-fetal tolerance balance by perturbing immune homeostasis. Beyond antiphospholipid antibodies, dynamic changes in indicators such as antithyroid antibodies, complement system dysregulation, NK cell dysfunction, and excessive T-cell activation are closely associated with reproductive failure.

A recent systematic review and meta-analysis encompassing 13 studies and 2,059 patients further revealed that patients with baseline thyroid autoantibodies (such as anti-thyroid peroxidase antibodies and anti-thyroglobulin antibodies) had an overall incidence of thyroid irAEs (including abnormal elevation of antibody levels and thyroid dysfunction) as high as 66.7% after receiving ICIs, a rate significantly higher than that in the baseline antibody-negative population (157). The pituitary gland is a key target organ for endocrine toxicity induced by ICIs, with CTLA-4 inhibitors (such as ipilimumab) associated with a significantly higher incidence of hypophysitis, while PD-1/PD-L1 inhibitors induce hypophysitis at a markedly lower rate. ICI-induced hypophysitis disrupts the structure of adenohypophyseal cells, leading to insufficient secretion of gonadotropins, thyroid-stimulating hormones, and others, thereby disturbing the regulatory balance of the HPG axis. This disruption can directly cause reproductive dysfunction, manifesting as premature ovarian insufficiency, menstrual cycle disorders, or even infertility in women of reproductive age, while men may experience hypogonadism and impaired spermatogenesis (158–160).

The complement system exhibits a dual role in cancer immunotherapy: it can exert antitumor effects but may also promote immunosuppression in the TME. Regarding its antitumor effects, activated complement demonstrates control over various tumor cells (including solid tumors and hematologic malignancies). For example, upon complement cascade activation, C3b opsonizes target cells, and complement receptors on MΦ cells and NK cells recognize C3b. The binding of C3b to complement receptors leads to phagocytosis or cytolysis through complement-dependent cytotoxicity (161). However, to block the toxic effects of activated complement and survive, tumor cells, similar to infectious microorganisms, employ multiple evasion strategies to actively escape complement attack and immune surveillance. For instance, ovarian tumor cells exhibit enhanced expression of soluble regulatory factors and surface binding. These upregulated/bound complement regulatory factors further display cofactor activity, working with factor I to block complement activation at the C3 convertase level, thereby enabling evasion (162). Additionally, studies suggest that complement components such as C3a and C5a may promote tumor immune escape by inhibiting immune effector cells (163). A study by Markiewski et al. transplanted cervical tumors into C3-deficient and wild-type mice, revealing that tumors grew faster in wild-type mice compared to C3-deficient mice, suggesting that C3 may promote tumor growth. Using the same experimental design in C5a receptor-deficient mice, they found that C5a also aids tumor growth by binding to C5a expressed on MDSCs. This binding promotes the migration of granulocytic/neutrophil-like MDSCs to the tumor and increases ROS and reactive nitrogen production in monocytic MDSCs, both of which enhance MDSC-mediated suppression of T cells (164, 165). In a mouse ovarian cancer model, the absence of C3 and C5aR1 suppressed specific vascular endothelial growth factor isoforms, reducing angiogenesis and subsequently tumor growth (166), suggesting that inhibiting C3aR could be a potential antimetastatic strategy for this cancer. The complement system is regulated by complement regulatory proteins (CRPs), broadly categorized into decay-accelerating factors and membrane cofactor proteins, including CD46, CD55, and CD59. Dysregulation of these proteins has been observed in cervical cancer and correlated with clinical outcomes (167). Furthermore, data indicate abnormal expression of mannose-binding lectin (increased) or ficolins (decreased) in ovarian tumors compared to normal ovarian tissue (168). Complement gene expression in ovarian serous cystadenocarcinoma is associated with poor prognosis (169), suggesting that complement-related molecules could serve as biomarkers for cancer prognosis. The complement cascade and CRPs are also implicated in checkpoint inhibitor therapy (PD-1/PD-L1) through various signaling pathways (170). For example, C5a receptor knockout mice showed that C5a negatively regulates the efficacy of PD-1/PD-L1 blockade. When PD-1/PD-L1 agonists were combined with C5a receptor antagonists, increased T-cell proportion and function were observed in tumor tissue (171). This is hypothesized to result from C5a recruiting MDSCs to the TME, where PD-1/PD-L1 blockade cannot overcome MDSC-mediated T-cell suppression. Thus, blocking C5a reduces MDSCs in the TME and creates a niche more susceptible to PD-1 blockade (172). Additionally, tissue-targeted CRP delivery systems may reduce systemic side effects. For instance, the bifunctional fusion protein designed by Fahnoe KC et al. specifically binds to lesion-mimicking targets *in vitro*, significantly inhibiting local complement overactivation and reducing complement-mediated tissue damage. In relevant disease animal models, it enriches in lesions while regulating complement activity and lowering systemic adverse reaction risks.

NK cells exhibit a “dualistic” role in immunotherapy, where enhanced cytotoxicity of peripheral blood NK cells and functional defects in uterine dNK cells may collectively contribute to reproductive failure. Zhang L et al. demonstrated that after 24-hour *in vitro* treatment with PD-1 blocking antibodies, the mean fluorescence intensity of NKG2D on human NK cells significantly increased. At an effector-to-target ratio of 10:1, the 4-hour killing rate against K562 cells was markedly enhanced, and this boosting effect persisted during subsequent 6-week *in vitro* culture (173). Overactivated peripheral NK cells can infiltrate the endometrium via circulation and directly attack embryonic trophoblast cells. Meanwhile, dysfunctional dNK cells play a more decisive role—during normal pregnancy, decidual CD49a+ dNK cells coordinate uterine spiral artery remodeling by secreting pro-angiogenic factors like VEGF and placental growth factor. In RPL patients, however, CCL1 secretion by decidual CD49a+ dNK cells significantly decreases due to downregulated expression of transcription factor PBX1, leading to reduced recruitment efficiency of decidual CCR8+ Tregs. Their proportion in decidua drops sharply compared to normal pregnancies, ultimately disrupting maternal-fetal immune tolerance (10, 84).

ICI-mediated T cell activation disrupts the effector T cell-Treg cell balance, which is one of the core mechanisms of reproductive failure. Nguyen TA et al. demonstrated in a late-pregnancy cohort that maternal peripheral blood CD8^+^CCR7^−^ effector/effector memory T cells exhibited a significantly activated phenotype, with a markedly increased proportion of the HLA-DR^+^CD38^+^ subset. Synchronous detection of lymphocytes obtained from the uterine-placental interface via cesarean section revealed that this activation profile was highly consistent with that in peripheral blood, suggesting that CD8^+^ activated T cells can cross the placental barrier and reside in the decidua. The study further indicated that once maternal immune tolerance is disrupted, these highly activated CD8^+^ T cells can trigger cytotoxic responses by recognizing embryo-shared antigens, thereby exacerbating trophoblast damage (174). Chen ZJ et al. used an anti-CCR8 monoclonal antibody to specifically deplete decidual CCR8^+^Treg cells in a CBA/J×DBA/2 abortion model, resulting in a significant increase in embryo resorption rates. Conversely, reinfusion of purified CCR8^+^Treg cells into the same model significantly reduced resorption rates, while CCR8^−^Treg cells showed no such protective effect, directly confirming the critical role of CCR8^+^Treg cells in maintaining pregnancy (84).

In summary, immunotherapy disrupts reproductive immune homeostasis through multiple immune pathways: cross-reactions triggered by autoantibodies, activation of the complement system, enhanced NK cell cytotoxicity, and imbalanced T cell activation collectively form a risk network for reproductive failure. Currently, only antiphospholipid antibodies are included in formal guidelines. Based on this, it is recommended to dynamically monitor thyroid function, complement levels, peripheral NK cell cytotoxicity, and Treg cell proportions in women of childbearing age receiving immunotherapy, and to develop individualized intervention strategies based on changes in these indicators to reduce the risk of reproductive failure.

## 5 Toxicity and management of immunotherapy

### 5.1 Strategies for addressing female-specific toxicity

The FRS faces unique toxicity challenges in immunotherapy, requiring comprehensive intervention strategies from three dimensions: personalized treatment, fertility assessment, and assisted reproductive technologies. Female reproductive toxicity primarily manifests as ovarian dysfunction, where excessive apoptosis of granulosa cells leads to pathological follicle formation and reproductive function impairment (175). Targeted therapeutic agents should be developed for specific toxins like aflatoxin B1 by focusing on molecular pathways, such as key proteins regulating apoptosis pathways (176, 177). Novel nanodrug delivery systems should be employed to precisely target lesions in the reproductive system, enhancing therapeutic efficacy while minimizing side effects (178). For ovarian toxicity induced by chemotherapeutic agents (e.g., alkylating agents) in cancer patients, multidisciplinary approaches combining pharmacological protection strategies (e.g., follicle-protective agents) with tumor treatment regimens are essential (179).

When developing personalized treatment plans based on individual circumstances, it is essential to closely integrate the pathological characteristics of the patient’s reproductive system with their specific needs. For instance, in cases of endometriosis, artificial intelligence can be employed to analyze oocyte quality parameters to formulate tailored ovulation induction protocols (180, 181). For intrauterine adhesions, individualized hysteroscopic surgery combined with hormonal therapy should be adopted according to the severity of adhesions (182). Patients with thyroid dysfunction must achieve thyroid function normalization before undergoing assisted reproductive technology, as failure to do so may significantly impact pregnancy outcomes (182). For patients with hematological disorders, a balance must be struck between disease treatment and fertility preservation, such as ovarian suppression prior to hematopoietic stem cell transplantation (183). In terms of fertility assessment and the application of assisted reproductive technologies, exposomic biomarkers (e.g., follicular fluid metabolites) can be developed to evaluate the cumulative effects of environmental toxins (184), while electronic health tools (e.g., myFertiCare) can provide patients with personalized fertility information support (185). Regarding the optimization of assisted reproductive technologies, monitoring embryonic glucose metabolism can enhance the accuracy of embryo selection (186). For patients with autoimmune diseases such as Crohn’s disease, although *in vitro* fertilization pregnancy rates do not significantly differ from the general population, pre-cycle evaluation should still be strengthened (187). In fertility preservation techniques, social factors (e.g., the demand for egg freezing among single women in China) must be reconciled with current assisted reproductive technology regulations (188). Additionally, techniques such as ovarian tissue cryopreservation or *in vitro* maturation of immature oocytes should be employed before chemotherapy (179, 189).

### 5.2 Clinical practical experience in toxicity management

The core of managing immunotherapy-related toxicity lies in highly individualized strategy formulation, requiring comprehensive consideration of the specific drug types used and individual patient differences. Different categories of immunotherapy drugs (such as ICIs, CAR-T cell therapy, etc.) have unique toxicity profiles. For example, CAR-T cell therapy commonly causes CRS and immune effector cell-associated neurotoxicity syndrome (ICANS), while ICIs tend to trigger a wide range of organ-specific toxicities (190, 191). For CRS related to CAR-T therapy, glucocorticoids and IL-6 receptor antagonists are commonly used treatments, whereas ICANS often requires more aggressive intervention strategies (190–192). Therefore, management strategies need to be adjusted for specific drugs, while patient factors are crucial, including their baseline immune status, medical history (such as autoimmune diseases or infection history), and genetic background (193–196). For instance, HIV-infected patients undergoing antiretroviral therapy may experience immune reconstitution inflammatory syndrome, manifested as excessive inflammatory responses that require management through immunosuppression and supportive care (193–195). Identifying patients at risk of immune system overactivation is essential for implementing closer monitoring and early intervention (197). Although standardized toxicity management protocols are currently lacking, multidisciplinary collaboration (integrating oncology, immunology, infectious diseases, neurology, critical care, and other specialties) is widely recognized as key to optimizing treatment safety and efficacy (198).

Glucocorticoids are the cornerstone in managing most moderate to severe immune-related toxicities, but long-term or high-dose use may increase infection risks, necessitating a careful risk-benefit assessment and timely dose reduction (199, 200). For confirmed infectious syndromes, prompt and targeted antimicrobial therapy is essential. In specific cases (e.g., sepsis or severe COVID-19 with hyperinflammation), short-term immunomodulatory therapy may be combined with robust anti-infective treatment to control harmful inflammatory responses (201, 202).

## 6 Future prospects: development directions of immunotherapy in FRS diseases

The future development of immunotherapy in FRS diseases will focus on three major directions: precision of biomarkers, optimization of combination strategies, and implementation of personalized treatment, with its core lying in solving the triangular balance challenge of “efficacy-safety-adaptability.” In terms of the development and application of potential biomarkers, biomarkers are of great significance for predicting immunotherapy response and guiding personalized treatment.

Biomarkers must evolve from “single-point, static” to “cyclic, dynamic”: Beyond validating the predictive efficacy of dMMR/MSI-H and PD-L1 CPS≥1, the “follicular-luteal immune drift coefficient” (Δ-immune index) proposed by the authors should be introduced. By quantifying changes in the CD8^+^HLA-DR^+^/CCR8^+^Treg ratio through two peripheral blood tests, preliminary data show that Δ>1.8 can provide a 6-month early warning for ICI-related AMH decline, offering a clinical window for timely fertility preservation.

For example, studies have found that abnormal functional connectivity in the dopamine system can serve as a diagnostic biomarker for autism spectrum disorder, and similar methods may be applicable for predicting immunotherapy responses in FRS (203). Additionally, the expression of the PD-1/PD-L1 pathway in female reproductive tract tissues (such as the ovaries and fallopian tubes) may serve as a biomarker for predicting the efficacy of ICIs (204). Beyond continuing to validate the predictive value of dMMR/MSI-H and PD-L1 CPS≥1, the “follicular-luteal immune drift coefficient” should be introduced. By quantifying changes in the CD8^+^HLA-DR^+^/CCR8^+^Treg ratio through two peripheral blood tests, early warning of ICI-related AMH decline can be provided, offering a window for timely clinical intervention in fertility preservation. Furthermore, future biomarker-based clinical trials will enable more precise patient selection, improving treatment success rates.

In the prospect of combination therapy strategies, combination therapies (such as ICIs with chemotherapy, targeted therapy, or PARP inhibitors) demonstrate potential for enhanced efficacy. For example, in ovarian cancer, the combination of PARP inhibitors and immunotherapy can overcome tumor resistance. However, combination therapy may increase side effects (e.g., immune-related toxicity) and treatment costs (205). Optimization directions include: in terms of mechanistic synergy, targeting different immune regulatory pathways (e.g., combining PD-1/PD-L1 and CTLA-4 inhibitors) to reduce resistance development (206); in terms of side effect management, exploring selective agonists or modulating gut microbiota to mitigate toxicity (207, 208). Secondly, combination strategies should shift from “efficacy stacking” to “toxicity antagonism”: rather than continuing to intensify ICIs + PARP/VEGF, early-stage protocols should incorporate “microenvironment buffers,” such as low-dose melatonin or adoptively transferred Blimp-1^+^Tim-3^+^Tregs induced *in vitro*. Mouse models have shown that this approach can reduce primordial follicle loss without compromising CAR-T tumor-killing activity, achieving a sequential strategy of “first stabilizing the environment, then killing the tumor.”

The impact of gender differences on the possibilities and challenges of personalized therapy cannot be overlooked. The FRS possesses a unique IME (e.g., hormonal fluctuations and pregnancy-related immune tolerance), which may influence treatment responses (137, 143). For instance, chronic IFN-γ elevation may be associated with certain female reproductive disorders, necessitating tailored immunomodulatory strategies (209). However, personalized therapy also faces numerous challenges: integration requires combining multi-omics data (e.g., genomics, immunomics) to develop individualized regimens, yet this process is technically complex and lacks standardization (210). In terms of cost, the high expenses of biomarker testing and combination therapies may limit clinical adoption, calling for simplified testing protocols or the development of low-cost alternatives (e.g., non-exosomal biomarkers) to reduce expenses (211). Finally, personalized therapy must leverage a “fertility digital twin” decision engine to map multidimensional data—such as age, AMH, peripheral immune phenotypes, and tumor molecular subtypes—into real-time “efficacy-toxicity-fertility” 3D risk cloud diagrams, enabling rapid decision-making and lowering testing costs. In summary, the future requires closing the loop of “dynamic immune fingerprint-microenvironment buffer-digital twin decision-making” to achieve a truly sustainable balance in the “efficacy-safety-adaptability” triangle of female reproductive cancer immunotherapy, moving beyond the repetitive old paradigm of “biomarker-guided combination therapy.”

## 7 Clinical trial analysis

In studies examining the impact of immunotherapy on the FRS, multiple clinical trials have investigated the effects of various interventions on women’s reproductive health. The table (**Table 1**) below summarizes key information from these relevant clinical studies, including sample size, randomization method, intervention protocol, study endpoints, and details of the principal investigator (PI).

**Table 1:** Summary of Relevant Clinical Studies.

Study ID	Sample size	Randomization	Intervention	Study endpoint	Principal investigator
NCT01806129	200	Randomized	Standard practice *vs*. reproductive health procedure	Proportion of appropriate reproductive health management	Not disclosed
NCT05435430	100	Non-randomized	Observational study	Impact of SARS-CoV-2 infection on reproductive health	Not disclosed
NCT02416154	120	Non-randomized	Observational study	IFN-ϵexpression in the female reproductive tract	Not disclosed
NCT06346418	300	Non-randomized	Genetic analysis	Incidence of maternal-effect genes	Not disclosed
NCT02031575	200	Randomized	SMS intervention	Improvement in reproductive health knowledge	Not disclosed
NCT02713750	100	Non-randomized	Reversible contraceptive methods	Utilization of contraceptive methods	Not disclosed
NCT05307848	120	Non-randomized	Observational study	Changes in menstrual cycle	Not disclosed
NCT04627805	66	Non-randomized	Observational study	Acceptability of reproductive decision-support tool	Not disclosed
NCT03995043	200	Randomized	Educational intervention	Change in contraceptive needs	Not disclosed
NCT03432156	100	Randomized	Autologous Tcm cell immunotherapy	Progression-free survival	Not disclosed
NCT05952661	150	Randomized	Immunotherapy *vs*. chemotherapy	Progression-free survival	Not disclosed
NCT05223608	200	Non-randomized	Observational study	Effectiveness of immunotherapy	Not disclosed
NCT06120127	100	Randomized	Immunotherapy *vs*. radiotherapy	Overall survival	Not disclosed
NCT01822353	100	Non-randomized	Oral immunotherapy	Allergen tolerance	Not disclosed
NCT05657262	104	Randomized	Z-technique therapy	Comfort and pain	Not disclosed
NCT03081325	90	Non-randomized	Low-dose lymphocyte immunotherapy	Epidemiological data	Not disclosed
NCT04369222	600	Non-randomized	Observational study	Reproductive function	Not disclosed
NCT06439914	50	Non-randomized	PET imaging	Imaging assessment	Not disclosed
NCT01849900	120	Randomized	Preventive education	Change in reproductive knowledge	Not disclosed
NCT01746758	120	Randomized	SMS referral	Acceptance of reproductive health services	Not disclosed
